# Innovative Breakthroughs for the Treatment of Advanced and Metastatic Synovial Sarcoma

**DOI:** 10.3390/cancers15153887

**Published:** 2023-07-30

**Authors:** Lorena Landuzzi, Maria Cristina Manara, Laura Pazzaglia, Pier-Luigi Lollini, Katia Scotlandi

**Affiliations:** 1 Experimental Oncology Laboratory, IRCCS Istituto Ortopedico Rizzoli, 40136 Bologna, Italy; mariacristina.manara@ior.it (M.C.M.); laura.pazzaglia@ior.it (L.P.); 2 Laboratory of Immunology and Biology of Metastasis, Department of Medical and Surgical Sciences (DIMEC), University of Bologna, 40126 Bologna, Italy; pierluigi.lollini@unibo.it

**Keywords:** synovial sarcoma, SS18::SSX fusion oncogene, soft tissue sarcoma, metastatic disease, trabectedin, pazopanib, PROTAC degrader, immune check point inhibitors, tertiary lymphoid structures, engineered T-cell receptor, T-cell adoptive transfer

## Abstract

**Simple Summary:**

Synovial sarcoma (SyS) is a rare malignant soft tissue sarcoma bearing the chromosomal translocation t(X;18), which encodes the fusion oncoprotein SS18::SSX. More than 80% of the patients, mainly young in age, are initially diagnosed with localized disease with a 5-year survival rate of 70–80%. Metastatic relapse occurs in 50% of the cases. Advanced, unresectable, or metastatic disease shows a poor prognosis with a 5-year survival rate below 10%, representing an urgent clinical issue. This review will focus on: (i) current front-line therapies; (ii) alternative treatments in second line and beyond settings; and (iii) new epigenetic and immunological strategies. The improved understanding of the SyS molecular biology coupled with the recent development of innovative technologies, such as proteolysis targeting chimera (PROTAC) protein degraders or adoptive transfer of engineered immune cells, is offering new promising tools. Clinical trial results underline the need for accurate patient selection based on genetic and tumor immune microenvironment signatures.

**Abstract:**

Synovial sarcoma (SyS) is a rare aggressive soft tissue sarcoma carrying the chromosomal translocation t(X;18), encoding the fusion transcript SS18::SSX. The fusion oncoprotein interacts with both BAF enhancer complexes and polycomb repressor complexes, resulting in genome-wide epigenetic perturbations and a unique altered genetic signature. Over 80% of the patients are initially diagnosed with localized disease and have a 5-year survival rate of 70–80%, but metastatic relapse occurs in 50% of the cases. Advanced, unresectable, or metastatic disease has a 5-year survival rate below 10%, representing a critical issue. This review summarizes the molecular mechanisms behind SyS and illustrates current treatments in front line, second line, and beyond settings. We analyze the use of immune check point inhibitors (ICI) in SyS that do not behave as an ICI-sensitive tumor, claiming the need for predictive genetic signatures and tumor immune microenvironment biomarkers. We highlight the clinical translation of innovative technologies, such as proteolysis targeting chimera (PROTAC) protein degraders or adoptive transfer of engineered immune cells. Adoptive cell transfer of engineered T-cell receptor cells targeting selected cancer/testis antigens has shown promising results against metastatic SyS in early clinical trials and further improvements are awaited from refinements involving immune cell engineering and tumor immune microenvironment enhancement.

## 1. Introduction

Synovial sarcomas (SyS) are rare malignant tumors representing 5–10% of all soft tissue sarcomas (STS). SyS can occur at any age but at least one third of the patients are children, adolescents and young adults and the median age of onset is around 35–40 years [1,2,3,4]. SyS mainly arises in the extremities but can as well occur elsewhere and, despite the name, it does not strictly derive from synovial tissue. Different morphologies can be identified: the monophasic type displays mesenchymal spindle cells only, the biphasic type presents both epithelial and spindle cells, and the poorly differentiated type shows round small cells and is associated with the worst prognosis [5].

SyS are identified by the presence of the chromosomal translocation t(X;18) giving rise to the SS18::SSX fusion oncogene and to the corresponding fusion protein. The pathognomonic translocation can be detected by fluorescence in situ hybridization (FISH), or the fusion transcripts can be identified by reverse-transcriptase polymerase chain reaction (RT–PCR). Of note, a rabbit monoclonal antibody (clone E9X9V) that specifically detects the fusion epitope of the endogenous human SS18::SSX protein was recently validated for immunohistochemistry and was made commercially available, simplifying the diagnosis of SyS and related molecular biology investigations [6].

Several in vitro and in vivo experimental models, including conditional genetically engineered mouse models (GEMM) carrying the SS18::SSX translocation and developing SyS and SyS patient-derived xenografts (PDX), have been developed and preclinical studies have highlighted many potential therapeutic targets [7]. Thanks to these models, a more comprehensive understanding of the mechanisms of action of the fusion gene [8], of the role of tumor microenvironment, and of tumor antigen expression is now opening the way to the design of innovative epigenetic, targeted, or immunological therapies, which can hopefully bring about significant changes in the mainstay of advanced SyS treatment.

This review will briefly summarize the new insights into the molecular biology of SyS and will focus on current treatment options and emerging new epigenetic and immunological strategies for the treatment of advanced metastatic SyS.

## 2. SyS Molecular Biology

The specific chromosomal translocation t(X;18) (p11.2; q11.2) generates the SS18::SSX fusion gene (including SS18::SSX1, SS18::SSX2, or rarely SS18::SSX4). In contrast to other translocated proteins, the SS18::SSX fusion protein lacks a DNA binding domain and is not a direct transcription factor. It is derived from the substitution of SS18 carboxy-terminal residues with an SSX carboxy-terminal tail [9]. Native SS18 is a member of the multimeric structure switch/sucrose non-fermentable (SWI/SNF) chromatin-remodeling complex family, also known as BRG1/BRM-associated factor (BAF) complexes, which are multimeric structures assembled of at least 29 different proteins. Depending on the distinct subunit composition, three final complexes can be recognized: canonical BAF (CBAF), polybromo-BAF (PBAF), and non-canonical BAF (ncBAF or GBAF). BAF complexes facilitate transcription by increasing chromatin accessibility at promoter and enhancer regions with different functional roles: CBAF localizes to enhancers and, among other roles, regulates self-renewal in embryonic cells; PBAF plays a role in differentiation and in maintaining genomic integrity during mitosis; and GBAF is mainly involved in maintaining stem cell pluripotency [10]. SS18 is a subunit of the CBAF and GBAF complexes [11]. On the contrary, SSX1/2 belong to a family of transcriptional repressors co-localizing with polycomb group proteins (PcG). An updated description of this increasingly complex family of proteins is offered in the recent review by Piunti and Shilatifard [12]. In particular, the SSX carboxy-terminal tail interacts with core proteins in the polycomb repressive complexes 1 (PCR1) and 2 (PCR2) associated with transcription silencing in mammalian development. SSX1/2 proteins interact with the BMI1 and RING1A proteins in PCR1, which is a histone ubiquitin ligase that targets Lys118 and Lys119 of histone H2A (H2AK118ub and H2AK119ub) through E3 ubiquitin ligase activity, and with SUZ12, EZH2, and EED proteins in PRC2, which is an histone methyltransferase responsible for monomethylated, dimethylated, and trimethylated histone H3 Lys27 (H3K27me1, H3K27me2, and H3K27me3) [12]. The chimeric oncoprotein thus assembles two components with opposing effects on chromatin regulation. The exact oncogenic mechanisms are still not fully understood. One hypothesis is that the SS18::SSX fusion protein replaces SS18 in CBAF and GBAF complexes resulting in the redirection of the newly constituted BAF complexes from the promoter and enhancer regions to PcG domains to relieve PRC2–mediated repression of bivalent target promoters [13]. The de novo, gain-of-function targeting of BAF complexes activates the unique SyS gene expression signature [13], generates multiple epigenetic deregulations, and triggers multiple oncogenic signaling networks [14]. In addition, these fusion genes can directly or indirectly regulate histone deacetylase HDAC1/2 activity [9,15]. Moreover, a repressive role for the SS18::SSX fusion oncoprotein has also been proposed, which is mediated by the PRC2 recruitment to activating transcription factor-2 (ATF2) target genes [9,16].

**Figure 1:**
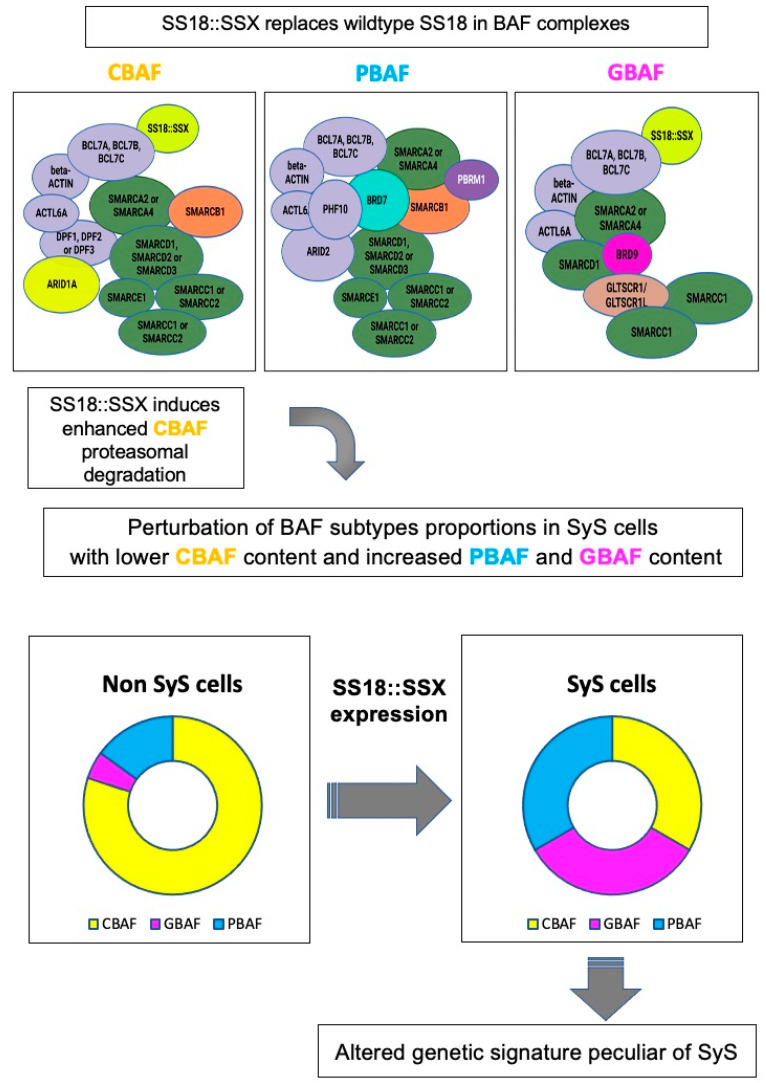
In SyS cells, the fusion oncoprotein SS18::SSX replaces the native SS18 subunit in CBAF and GBAF complexes and is supposed to induce increased proteasomal degradation of CBAF complexes. As a consequence, in SyS cells, the relative abundance of the different BAF complexes subtypes is modified in favor of PBAF and GBAF, which have different functional roles and chromatin distribution; therefore, this determines the acquisition of the unique SyS genetic signature (modified from Li et al. and Nacev et al. [8,10]) (created with Biorender.com accessed on 25 July 2023).

Recently Li and coworkers [8] demonstrated that incorporation of SS18::SSX into CBAF complexes leads to their degradation, inducing a strong imbalance in BAF complexes fractional abundance in SyS cells with downregulation of CBAF and upregulation of GBAF and PBAF. Targeting of GBAF was suggested as a possible therapeutic strategy in SyS, and drugs degrading BRD9, which is exclusively present in the GBAF complex, were able to inhibit SyS growth in experimental models [8]. Figure 1 illustrates the changes in the BAF complex balance caused by the SS18::SSX fusion oncoprotein in SyS cells and the related acquisition of the unique SyS genetic signature.

Overall, like many other translocated sarcomas, SyS are characterized by a simple and stable karyotype with few secondary genetic alterations. TP53 is rarely mutated in SyS, while copy number gains in the p53 suppressing oncogene MDM2 are slightly more common, as well as alterations within AKT/PTEN, with frequent activation of the AKT/mTOR and Wnt signaling pathways [17,18]. Most gene expression profiling studies on SyS show a high expression of mediators involved in early embryogenesis, including the Wnt, NOTCH, Hedgehog, FGF, and BMP pathways [19]. In addition, through sequencing approaches, mutations in PTEN, CTNNB1, and APC have been identified [20,21,22]. The high expression of neural or chondrocyte lineage markers and of selected cancer/testis antigens (CTA), such as New York esophageal squamous cell carcinoma 1 (NY-ESO-1), melanoma antigen A4 (MAGE-A4), or preferentially expressed antigen in melanoma (PRAME), are common in SyS [23]. As described later, most of this evidence is now being exploited for therapeutic purposes.

## 3. SyS Current Therapies

Surgery, with either neoadjuvant or adjuvant radiotherapy, and chemotherapy represent the standard of care for local and advanced disease, respectively. Tumor size, tumor location, distant metastasis, age of patients under 20 years old, complete resection surgery, and radiotherapy are the main prognostic factors. Most of the patients, over 80%, are initially diagnosed with localized disease [24] and show a 5-year survival rate of 70–80%. Development of metastases, mainly involving the lung, is expected in 50% of the cases. In advanced metastatic disease, the survival rate at 5 years is dismal, below 10%, and novel therapeutic approaches are strongly needed to improve outcomes.

### 3.1. SyS Standard of Care in the Front-Line Setting

Systemic treatment represents the mainstay for the management of patients with advanced unresectable or metastatic SyS. Anthracycline and ifosfamide based chemotherapy constitute the most common therapy in the first line setting [25,26]. Compared to other STS, SyS is characterized by a higher sensitivity to chemotherapy. Based on a pooled review of 15 EORTC (European organization for research and treatment of cancer) soft tissue and bone sarcoma group clinical trials, front line treatment with cytotoxic chemotherapy in metastatic disease achieved a response rate of 27.8% in SyS compared to 18.8% in other STS [4]. The median overall survival (OS) for metastatic SyS was 15–20 months and complete response (CR) was very rare; therefore, advanced metastatic SyS still represents a highly unmet clinical issue. Other cytotoxic drugs, such as gemcitabine, docetaxel, and dacarbazine, are permitted for all STS, but with limited utility in SyS [26]. Even eribulin, a tubulin-targeting drug, showed only some clinical benefit but no significant activity in SyS patients [27].

### 3.2. SyS Second Line Setting and Beyond

The treatment options for locally advanced, unresectable, and metastatic SyS are far from satisfactory and only a minority of patients achieve objective responses under conventional chemotherapy. After failure of the first line treatment, further therapies are unable to provide any potential for cure and most of the time can only offer a short-term clinical benefit. A second line standard of care for SyS patients, as well for STS, has not yet been established, and even higher variability exists in third line and beyond treatments. Second line treatments in SyS are mainly represented by trabectedin and pazopanib, with some differences for the two drugs in approval history by US and EU regulatory agencies [25], and by high-dose continuous infusion of ifosfamide [28]. In the absence of a recognized second line standard of care, additional predictive parameters are strongly needed to support clinical decisions.

#### 3.2.1. Trabectedin

Trabectedin, also known as Ecteinascidin-743 (ET-743), is an alkylating agent initially discovered in the tunicate Ecteinascidia turbinate and is now produced synthetically. Trabectedin covalently binds to the minor groove of DNA, inducing structural changes, altering DNA repair mechanisms, and interfering with the DNA binding of minor groove-interacting transcriptional factors, such as nuclear factor-Y, which regulate genes involved in cell cycle control [29]. Among the many mechanisms of action, trabectedin exerts indirect anti-inflammatory and anti-angiogenic activities by acting on tumor-associated macrophages. Relevant also for the increasing use of immunotherapy is the observation that trabectedin not only kills cancer cells and increases the expression of tumor neoantigens for immune recognition; it also exerts a peculiar activity on the monocyte–macrophage compartment by reducing the number of M2 macrophages with tumor promoting activity in the tumor microenvironment.

Currently, trabectedin is used in metastatic STS after progression on first line anthracycline-based chemotherapy. A meta-analysis of real world and clinical trial studies revealed that trabectedin is one of the most used treatments for metastatic SyS in second line and beyond therapies, being administered in approximately 30% of the patients [25]. Several studies have reported improved progression free survival (PFS) in translocation-associated STS, including SyS, after treatment with trabectedin. A retrospective analysis analyzing translocation-related sarcomas reported a 22% PFS at 6 months in a cohort of 45 SyS patients receiving trabectedin [30]. Similar results were described in a different retrospective study analyzing 61 SyS patients treated with trabectedin, which showed a 6-month PFS of 23% and an overall response rate (ORR) of 15% [31]. In a randomized prospective trial analyzing trabectedin compared to best supportive care (BSC) in 76 patients with translocation-related sarcomas, including a small number of SyS, who had previously failed an anthracycline-based therapy, a significantly better response rate of 8% vs. 0% was demonstrated in the entire cohort as compared to BSC with a longer median PFS of 5.6 vs. 0.9 months [32,33]. Finally, a meta-analysis across nine real world and clinical trial studies reported an ORR of 12.3%, a median PFS of 3.4 months, and median OS of 10.4 months for SyS patients treated with trabectedin in a second line setting, highlighting the need of further improvement [25].

Of note, the systemic inflammatory response and related inflammatory indexes, such as high neutrophil-to-lymphocyte ratio (NLR) and low lymphocyte-to-monocyte ratio (LMR), were found to be significantly associated with poor prognosis in SyS [34,35]. In a retrospective study, patients with high LMR and treated with trabectedin showed a better PFS compared to patients with a high pre-treatment monocyte level receiving other therapies [36], suggesting that trabectedin represents a valuable treatment in patients with high LMR.

#### 3.2.2. Pazopanib and Tyrosine Kinase Inhibitors

Pazopanib is a multitargeted tyrosine kinase inhibitor (TKI) of vascular endothelial growth factor receptors 1, 2, and 3 (VEGFR1-2-3), platelet-derived growth factor receptor (PDGFR), c-kit, and fibroblast growth factor receptors (FGFR), with potent anti-angiogenic properties. Pazopanib has shown anti-tumor activity in SyS [33], and in 2012, it received US and EU approval for the treatment of non-adipocytic metastatic STS after failure of first line anthracycline-based chemotherapy [25]. In addition to retrospective studies, the phase III placebo-controlled PALETTE study showed a significant improvement in the median PFS in pazopanib treated STS patients of 4.6 vs. 1.6 months, but with no significant improvement in OS. In the 38 SyS patients that were included in the PALETTE study, pazopanib showed an improved median PFS of 4.1 vs. 1.0 months with placebo. Recently, a meta-analysis of the literature, focused on metastatic SyS treatment, analyzing seven studies, both real world and clinical trials, using pazopanib reported a median OS of 10.3 months, a median PFS of 5.3 months, and an ORR of 18.9% [25]. Similar activity was reported also for regorafenib, another TKI that inhibits VEGFR1-2-3, and tumor cell TK (RET, KIT, PDGFR, and Raf). In the randomized placebo controlled REGOSARC trial, regorafenib showed a median PFS of 5.6 months in the SyS cohort versus 1.0 month in the placebo arm [37,38].

## 4. SyS Innovative Therapies and Ongoing Clinical Trials

In contrast with oncogenes having tyrosine kinase activity, the properties of the SyS fusion oncoprotein, involved in transcriptional and chromatin remodeling functions, are more difficult to disrupt. Inhibiting its function, blocking its interactions, or triggering its selective degradation remain the greatest challenges for effective therapies. However, the improved understanding of the molecular mechanisms behind SyS, knowledge on tumor antigen expression and the immune microenvironment in SyS, and the parallel development of innovative technologies for the design of new molecules, such as proteolysis targeting chimera (PROTAC) protein degraders or for immune cell molecular engineering, are now offering new opportunities for the treatment of advanced metastatic SyS [39,40].

### 4.1. Epigenetic Modifiers

The fusion oncogene SS18::SSX plays a driver role in SyS by inducing broad epigenetic dysregulation involving multiple mechanisms. Histone acetylation is one of the key factors in the epigenetic regulation of gene expression. Histone deacetylase (HDAC) inhibitors have shown efficacy against SyS in pre-clinical studies and on these bases, entered clinical assessment. A phase II trial (ClinicalTrials.gov Identifier: NCT00918489) reported a median PFS of 3.2 months, and a median OS of 12.3 months with no objective responses after treatment with vorinostat (HDAC inhibitor) in 40 pretreated STS patients, including three SyS patients [41]. Similarly, another phase II trial (ClinicalTrials.gov Identifier: NCT01136499) evaluating the efficacy of panobinostat, a potent HDAC inhibitor, in patients with STS, including six SyS patients, also showed limited activity with no objective response in SyS [42].

The enhancer of zeste homolog 2 (EZH2) is a histone lysine N-methyltransferase enzyme and represents the functional enzymatic subunit of PCR2 having altered activity in SyS. A phase II basket study (ClinicalTrials.gov Identifier: NCT02601950) evaluated the activity of an EZH2 inhibitor, tazemetostat, in STS and showed no objective responses and a median PFS of 5 months in the 33 SyS patients enrolled [43]. Collectively, these data suggest that targeting EZH2 or HDAC is not sufficient to revert epigenetic alteration and tumor progression in advanced metastatic SyS.

### 4.2. BRD9 Degraders

Bromodomain-containing protein 9 (BRD9) is a non-BET bromodomain protein that uniquely participates in GBAF complexes, which are highly represented in SyS. For this reason, BRD9 inhibition and/or degradation have been recognized as potential strategies for the treatment of SyS. The PROTAC technology has been applied to target BRD9. PROTAC molecules are represented by heterobifunctional small molecules consisting of two ligands joined by a linker: one ligand binds to the target protein and the other recruits an E3 ubiquitin ligase. Ubiquitylation of the target protein drives its degradation by the ubiquitin proteasome system, and after degradation, the PROTAC molecule is recycled to repeat the process [44]. Thanks to PROTAC technology, some BRD9 chemical degraders that bridge the BRD9 bromodomain and E3 ubiquitin ligase complexes were developed. Degradation of BRD9 inhibited SyS tumor progression in a mouse model [14]. Therefore, BRD9 inhibition and/or degradation represents a potential strategy for the treatment of SyS. CFT8634 (ClinicalTrials.gov Identifier: NCT05355753) is an oral heterobifunctional degrader that bridges BRD9 with E3 ligase, causing ubiquitination and proteasomal degradation of BRD9. FHD-609 is an intravenous BRD9 degrader that bridges BRD9 with the E3 ubiquitin ligase substrate cereblon (CRBN) E3 that leads to proteasomal degradation. These therapies are under initial evaluation in phase I clinical trials, which are now recruiting patients with advanced SyS who failed prior anti-cancer therapies [44,45].

## 5. Immunotherapy in Advanced Metastatic SyS: Selected Use of Immune Check Point Inhibitors and Adoptive Transfer of Engineered Immune Effectors

SyS belongs to the category of translocated sarcomas with a low mutational burden. Since SyS usually displays poor immune infiltration, it is considered an immune-cold tumor. Interestingly, single cell RNA sequencing in SyS identified a subgroup of malignant cells (cycling and poorly differentiated cells) expressing the core oncogenic program of SyS. These cells were mainly localized in immune-deprived niches in situ and were predictive of poor clinical outcomes in two independent cohorts. The core oncogenic program is controlled by the SS18::SSX fusion, is involved in immune cell evasion and is able to activate intrinsic oncogenic mechanisms that actively repress SyS infiltration of immune cells [46]. Targeting the mechanisms of immune evasion is an open challenge that has been approached from multiple directions. With this intent, several clinical trials were set up to investigate the anti-tumor efficacy of immune checkpoint inhibitors (ICI) or the efficacy of the adoptive transfer of modified immune effectors in advanced metastatic SyS.

### 5.1. Immune Checkpoint Inhibitors in SyS

ICIs against PD-1, PD-L1, and CTLA-4, and combinations, have consistently improved patient survival across several aggressive tumor entities and have entered into the adjuvant, and possibly next neoadjuvant, management of patients with various advanced solid tumors such as melanoma, non-small-cell lung cancer, bladder cancer, renal cell carcinoma, and colon carcinoma [47,48,49].

The activity of ICIs in sarcomas is more challenging and only recently, in December 2022, atezolizumab (an anti-PD-L1 antibody) received FDA approval for advanced alveolar soft part sarcoma (ASPS), for which an overall response rate of 24% and a 42% duration of response over 12 months was observed (https://www.fda.gov/drugs/resources-information-approved-drugs/fda-grants-approval-atezolizumab-alveolar-soft-part-sarcoma accessed on 13 April 2023) (ClinicalTrials.gov Identifier: NCT03141684). Indeed, a pooled analysis of clinical trials investigating ICIs in STS [50] highlighted that ASPS and undifferentiated pleomorphic sarcoma (UPS) exhibited the highest response rates. The anti-tumor efficacy of ICIs in other STS histotypes is more limited and SyS, although poorly represented across multiple trial cohorts, did not emerge as an ICI-sensitive tumor.

SARC028 (NCT02301039) was one of the pioneering clinical trials with ICIs in sarcoma patients, but it did not meet the primary endpoint of overall response [51]. Treatment with pembrolizumab, anti-PD-1 monotherapy, showed limited activity only in UPS and dedifferentiated liposarcoma (DDLPS) [52]. The outcome of SyS patients, with one partial response and one stable disease out of ten patients, was below expectation [51]. Similarly, a phase 2 trial using ipilimumab (anti-CTLA4 antibody) to treat SyS expressing the NY-ESO-1 antigen enrolled only six patients and was terminated prematurely because of the complete absence of activity [53]. Even in a recent phase II clinical trial (ClinicalTrials.gov Identifier: NCT02815995) aimed at evaluating the combined treatment with durvalumab, an anti-PD-L1, and tremelimumab, an anti-CTLA-4, across multiple metastatic sarcoma subtypes [54], the enrollment to SyS cohorts was halted after the initial assessment did not show robust responses, with four out of five SyS patients presenting disease progression. An additional study using atezolizumab (anti-PD-L1 antibody) as a single agent or in combination with a vaccination protocol against NY-ESO-1 antigen in patients with advanced or metastatic SyS or myxoid liposarcoma (ClinicalTrials.gov Identifier: NCT02609984) showed that in the atezolizumab monotherapy arm, there was an absence of overall response but some clinical utility with stable disease in 34.5% of SyS patients, and a global 24-month OS rate of 40%, while no significant improvement was achieved in the combination arm [55]. In a different study, the combination of bempegaldesleukin, a PEGylated recombinant human interleukin-2 designed to induce activation and proliferation of CD8 T-cells and NK cells, with nivolumab (anti-PD1) did not improve the efficacy in sarcomas, including a small cohort of SyS (ClinicalTrials.gov Identifier: NCT03282344) [56].

Even hyperprogressive disease (HPD), a sudden increase in the tumor growth rate and disease progression, was observed; a pooled analysis of 134 STS patients of four prospective studies using ICIs reported HPD in 11% of patients across all histotypes, including SyS [57,58]. No tumor immune microenvironment differences, nor genomic alteration emerged as predictive factors for HPD risk in advanced sarcoma patients.

Overall, these results suggest that ICIs may lack a major therapeutic impact in SyS. Further studies have been proposed combining chemotherapy or targeted therapies with ICIs. Interesting results were obtained by a phase I/II clinical trial that explored the safety and efficacy of trabectedin, in addition to ipilimumab (anti-CTLA4) and nivolumab (anti-PD-1), as the first line treatment in advanced metastatic STS (ClinicalTrials.gov Identifier: NCT03138161). This study reported an ORR of 19.5% in metastatic STS. The cohort of the phase II study included five SyS patients that were among the best responders obtaining one complete response, two partial responses, and two stable diseases. Further randomized phase III studies are required to establish the value of the combination of a trabectedin and ICI regimen vs. doxorubicin plus ifosfamide as the first line treatment of advanced SyS and STS [59].

Additional studies potentially including SyS have been planned, for example, pembrolizumab combined with Lenvatinib, a multiple kinase inhibitor of VEGFR1-2-3, FGFR1-2-3-4, PDGFR alpha, c-kit, and Ret in the NCT04784247 trial; or nivolumab (anti-PD1) plus gemcitabine/doxorubicin/docetaxel (GALLANT, NCT04535713); or sintilimab (anti-PD-1) plus doxorubicin/ifosfamide (NCT04356872); or camrelizumab (anti-PD-1) plus doxorubicin/ifosfamide (NCT04606108) [60]. Radiation therapy is another local therapy able to increase tumor immunogenicity and activate the immune microenvironment in tumors. Several ongoing trials are aimed at evaluating the effect of radiation in addition to ICI [60].

Results of these studies will further shed light on the utility of ICIs in SyS, but true improvements will probably only come from a better selection of patients and the greatest challenge will remain a higher ability to predict the patients most likely to benefit from ICI treatment. Of note, in most of these studies, PD-L1 expression was not correlated with treatment outcomes [51,59]. Even a low tumor mutational burden (TMB) in sarcomas does not predict a lack of response to immunotherapy, and the limited utility of the TMB as a biomarker in sarcomas is best exemplified by ASPS, which despite a low TMB, can respond to ICIs [58]. Overall, a more in-depth analysis of the different parameters is needed.

#### Biomarkers of the Immune Tumor Microenvironment

Successful tumor immunotherapy requires the accurate identification of which tumor immune microenvironment conditions are associated with a better response. Petitprez and collaborators [61] provided an immune profile-based categorization of STS with prognostic relevance. Based on consensus clustering of the immune cell abundance and immune function gene signature scores, five distinct sarcoma immune classes (SIC) were identified: (A) immune-desert, characterized by the lowest expression of gene signatures related to immune cells and vasculature; (B) immune-low; (C) highly vascularized, showing high expression of endothelial cell-related genes; (D) heterogeneous immune-high, with high expression of genes specific to immune populations, such as T-cells, CD8+ T-cells, and natural killer (NK) cells; and (E) immune-high with the highest B cell signature. At the in-situ evaluation, the SIC E was the only group characterized by the presence of intratumoral tertiary lymphoid structures (TLS), constituted by ectopic aggregations of B and T lymphocytes and follicular dendritic cells. TLS can be found in non-lymphoid tissues, at the sites of chronic inflammation, including tumors. Interestingly, the expression of major histocompatibility complex (MHC) class I and the expression of genes associated with T-cell/myeloid cell chemotaxis and activation were high in SICs D and E, intermediate in SICs B and C, and very low in SIC A. Of note, the presence of TLS and the SIC E subtype was found across all STS histotypes. The analysis of the 58 SyS patients in the cohort of the France Sarcoma Group (FSG) indicated that approximately 20% of patients belong to the SIC E “immune and TLS high” profile; around 50% of the patients belong to immune poor profiles, with 20% in the SIC A “immune desert” and 30% int he SIC B “immune low” profile. Analyzing different cohorts of STS patients (TCGA SARC and GSE21050, both including leiomyosarcoma (LMS), undifferentiated pleomorphic sarcoma (UPS), dedifferentiated liposarcoma (DDLPS), and FSG, including SyS, gastrointestinal stromal tumor (GIST), and myxoid liposarcoma), they found that patients with SIC A (immune desert) exhibited a significantly shorter overall survival compared with SIC E patients. A high B cell signature was the strongest positive prognostic factor even in the context of high or low CD8+ T-cell contents. When examining if SICs can predict patient response to ICIs, they analyzed the phase 2 clinical trial SARC028 (including LMS, UPS, and DDLPS), which evaluated the efficacy of pembrolizumab, an anti-PD-1 monoclonal antibody, in patients with metastatic STS. SIC E tumors were associated with the highest response rate and improved PFS in comparison with patients with SIC A or B tumors. Based on these analyses, they identified a group of patients with a better response to anti-PD1 therapy marked by B cells and TLSs. The authors hypothesized that treatment with ICIs may allow effective anti-tumor immunity in B-cell-high and TLS-rich tumors, and patients expected to respond could be better identified on these bases [61].

Of note, an additional cohort, enrolling 30 STS patients selected for the presence of TLSs, in the PEMBROSARC study (ClinicalTrials.gov Identifier: NCT02406781, a phase 2 study of pembrolizumab combined with low-dose cyclophosphamide in patients with advanced STS), fully confirmed this hypothesis. While it was previously demonstrated that, in an unselected population, the clinical benefit deriving from the use of ICIs was very limited, and the presence of TLS strongly correlated with the clinical response. The authors report that both the response rates and PFS were significantly higher in the TLS-enriched cohort than in the previous all-patient cohorts of the PEMBROSARC study (30% versus 2% and 4.9 versus 1.5 months, respectively) [62]. Post-hoc analyses revealed that the abundance of intratumoral plasma cells was significantly associated with improved outcome, while TLSs from non-responder patients were significantly more enriched in regulatory T-cells. These results indicate that TLS presence, in advanced STS, is a meaningful predictive biomarker of ICI response and can improve patients’ selection for ICI therapies. Moreover, two randomized clinical trials evaluating the impact of treatment with ICIs versus standard chemotherapy in patients with TLS-positive sarcomas in the neoadjuvant setting (NCT04968106) and metastatic setting (NCT04874311) were proposed [62]. The PEMBROSARC study did not include any SyS patients and further investigation in TLS-rich SyS patients is awaited.

An additional immune-related gene (IRG) signature settled on the expression of 14 genes based on the immune cell abundance score and the weighted gene co-expression network analysis highlighted a protective role for the immuno-infiltration score, several T-cell subtypes, and NK cell abundance. The 14-IRG signature was able to identify high risk patients across seven published datasets of various sarcoma subtype cohorts, including GEO: GSE40025 listing 86 SyS patients, and showed a high potential for the prediction of survival outcomes, as well as of the immunotherapy response, offering new tools for clinical decision making [63].

In a different study on STS patients, including SyS, resistance to ICIs was correlated with the upregulation of mesenchymal transition and of Hedgehog signaling pathway expression. The Hedgehog signaling pathway has been shown to drive tumor growth through several activities, which include enhanced immunosuppressive mechanisms. In a trial on STS, the presence of CD8+ T-cells and reduced expression of the Hedgehog signaling pathway led to the best clinical outcome; therefore, the relevance and the targeting of Hedgehog signaling in the immune microenvironment should be further investigated in STS and SyS [56].

### 5.2. Adoptive T-Cell-Based Cancer Immunotherapy Targeting Cancer/Testis Antigens in SyS

Cancer/testis antigens (CTAs) are a large family of antigens highly expressed in the testis and in transformed cancer cells, but not in somatic normal cells. Because of the wide epigenetic dysregulation, SyS displays multiple intracellular cancer/testis antigens that are presented on the cell surface in the contest of human MHC human leukocyte antigen (HLA) molecules and can potentially be targeted by the adoptive transfer of antigen-directed T-cell receptor-transduced T (TCR-T)-cells. Unlike CAR T-cells, which recognize cell surface antigens independently of HLA haplotype and expression, TCR-T-cells recognize an antigen presented by an HLA molecule; therefore, they depend on HLA for target recognition and activation. Autologous T-cells (CD4+ and CD8+) recovered from patients after leukapheresis can be retrovirally transduced, with a self-inactivating lentivirus vector, to encode a specific peptide enhanced affinity receptor (SPEAR) TCR recognizing, with high-affinity, a CTA peptide in a complex with selected HLA-A*02 alleles (HLA-A*02:01, HLA-A*02:05, and HLA-A*02:06), globally having around 30% frequency in the Caucasian population [64] and expanded in vitro before adoptive transfer. Adoptive cell transfers using engineered T-cell receptors specifically targeting CTA, such as NY-ESO-1, or MAGE-A4, or PRAME, have recently shown promising results for the treatment of metastatic SyS in several early-stage clinical trials restricted to selected HLA-A*02 genotypes. It is worth saying that, when investigating whether selected HLA-A*02 genotypes are predictive of the outcome in metastatic SyS, in a multivariable model, HLA-A*02 expression was not significantly associated with OS or response to standard treatments [64].

#### 5.2.1. NY-ESO-1

NY-ESO-1 is a highly immunogenic CTA aberrantly expressed in 60–80% of SyS [45,65,66], independently from the morphological or the translocation type [67]. In 2014, a pilot clinical trial (NCT00670748) on HLA-A*02-positive SyS patients with >50% expression of NY-ESO-1 demonstrated objective clinical responses in 11 metastatic SyS patients heavily pretreated out of 18 (61%) receiving retrovirally transduced NY-ESO-1 TCR-T-cells plus systemic IL2 [66]. More recently, a phase 1 clinical trial (ClinicalTrials.gov Identifier: NCT01343043), including 45 metastatic SyS patients treated with transgenic T-cells targeting NY-ESO-1 in the contest of HLA-A*02 alleles, demonstrated an ORR of 20–50% among four cohorts allocated between levels of NY-ESO-1 expression and lympho-depletion regimens [45,56,68,69,70]. The treatment had a controllable safety profile and a post-hoc analysis provided further outputs. The level of expression of NY-ESO-1 had no significant impact on response [70]. A direct correlation with response was found with the use of the standard lymphodepleting chemotherapy regimen (LDR) (fludarabine and cyclophosphamide), compared to reduced LDR prior to TCR-transduced T-cell infusion. Use of standard LDR appeared to create favorable conditions for T-cell proliferation and higher IL-15 levels pre-infusion. Standard LDR and the higher weight-normalized infused TCR-transduced effector memory CD8+ T-cell dose were associated with higher peak cell expansion, which was a marker of response. Compared to non-responders, responders had increased IFNγ, IL-6, and peak cell expansion suggesting activation of T-cells and modulation of the cytokine setting within the tumor microenvironment. Interestingly, analysis of tumor samples post-treatment showed decreased expression of macrophage genes, suggesting changes in the tumor microenvironment leading to reduced macrophage infiltration [70]. Of note, when analyzing mechanisms of resistance, it was observed that non-responders displayed elevated myeloid and macrophage infiltration, and tumor biopsies obtained at progression showed retainment of NY-ESO-1 expression but a decrease in HLA-A2 and genes involved in antigen presentation. Gyurdieva and collaborators [70] concluded that strategies able to upregulate HLA expression, in order to overcome low antigen presentation and to reduce recruitment of macrophages and myeloid cells, could improve the anti-tumor efficacy and have an important impact on patient response. Refinements of NY-ESO-1 engineered TCR treatments are currently being explored in further clinical trials. A phase II study (NCT03967223) is aimed at evaluating the anti-tumor efficacy of the first generation of NY-ESO-1 specific T-cell receptor engineered T-cells as a first line treatment in advanced metastatic, previously untreated, HLA-A*02-positive patients with NY-ESO-1-positive metastatic or unresectable SyS or myxoid/round cell liposarcoma (MRCLS), and as a second line treatment in patients with advanced SyS or MRCLS who have progressed after first line anthracycline-based chemotherapy. An additional phase II master protocol of three different next generation NY-ESO-1 T-cell products co-expressing the cluster of differentiation 8 (CD8) alpha cell surface receptor, or co-expressing the dominant-negative TGF-beta receptor type II (dnTGF-beta RII) cell surface receptor, or engineered using the epigenetically reprogrammed (Epi-R) manufacturing process, respectively, enhancing T-cell antigen binding, altering the tumor microenvironment, and improving the quality of T-cells used for treatment, has been proposed for the treatment of HLA-A*02 positive patients with NY-ESO-1-positive previously treated advanced SyS, MRCLS and metastatic non-small cell lung cancer (NSCLC) (NCT04526509).

The combination of NY-ESO-1-specific TCR-T-cells infused twice into HLA-matched SyS NY-ESO-1-positive patients and a lymph node-targeting nanoparticulate peptide vaccine containing the NY-ESO-1 epitope recognized by TCR-T-cells, without previous lymphodepletion, was tested in a small phase I clinical trial (Registration ID: JMA-IIA00346) including three SyS patients. Two partial responses and one progressive disease were reported [71].

To overcome the need for HLA matching or loss of HLA expression on tumor cells, new NY-ESO-1 artificial adjuvant vector cells (aAVC) are being developed. ASP0739 are human artificial adjuvant vector cells loaded with the CD1d ligand alpha-galactosylceramide and are modified to express NY-ESO-1, which potentially induced the cytotoxic T-cell mediated response against tumor cells expressing NY-ESO-1. A phase I/II trial has been designed to evaluate safety and anti-tumor efficacy of this aAVC product targeting NY-ESO-1 in combination with pembrolizumab (anti-PD-1) for the treatment of SyS, MRCLS, ovarian carcinoma, non-small cell lung cancer, and esophageal squamous cell carcinoma (NCT04939701) [45].

#### 5.2.2. MAGE-A4

MAGE-A4 is an additional CTA highly expressed in 82–88% of SyS [65,72]. Similar to NY-ESO-1, MAGE-A4 is an intracellular antigen processed and presented in peptide fragments in the contest of HLA molecules. It is recognized by TCR; therefore, it represents an interesting target for adoptive T-cell therapies using specific peptide enhanced affinity receptor (SPEAR) T-cell therapy. After extensive preclinical testing, the afamitresgene autoleucel (afami-cel) therapy, represented by autologous T-cell transduced, after leukapheresis, with a lentiviral vector to express a high-affinity TCR specific for a MAGE-A4 230–239 peptide, GVYDGREHTV, presented by HLA-A*02, and expanded in vitro [73], was proposed for clinical development.

Afami-cel (or ADP-A2M4) therapy was studied in a phase I multi-tumor trial aimed at evaluating safety and clinical activity of afami-cel in HLA-A*02+ patients with advanced metastatic MAGE-A4-expressing solid tumors across nine tumor types, including SyS (NCT03132922). Patients received prior cyclophosphamide and fludarabine lymphodepletion chemotherapy followed by afami-cel at escalating doses in each group. The ORR, represented by partial responses only, was 7/16 (44%) for SyS and 2/22 (9%) for all other tumor types. The disease control rate, including PR and SD, in SyS was 94% with a median PFS of 20.4 weeks and median OS of 58.1 weeks [74]. These results appear very promising in comparison with low OS at around 10 months, which was achieved with current second line standard-of-care therapies, including pazopanib and trabectedin [25]. Of note, all seven responding SyS patients had high tumor MAGE-A4 expression and were treated with high doses afami-cel (4.5–>9.5 × 10^9^). Cytokine release syndrome (CRS), managed with administration of anti-IL-6(R) monoclonal antibody, occurred in 55% of all patients and was more frequent in responders. When analyzing serum cytokines, peak serum IFNγ levels were significantly higher in responders than non-responders. The presence of afami-cel was detectable in 67% of tumor biopsies and in blood samples of all patients up to 18 months post-infusion. Tumor cell lysis ability was preserved over time.

Overall, afami-cel had an acceptable benefit–risk profile and appeared to be an effective therapy for patients with advanced metastatic SyS. The results of the phase I trial fostered the phase II SPEARHEAD-1 trial (ClinicalTrials.gov Identifier: NCT04044768) aimed at investigating the efficacy and safety of ADP-A2M4 in HLA-A*02 eligible and MAGE-A4 positive subjects with advanced metastatic or inoperable SyS or MRCLS [74]. Based on safety findings, for the phase II study, the lower LD chemotherapy regimen and the highest dose of afami-cel (1.0–10 × 10^9^ transduced cells) were selected. Results from the phase II trial were consistent with those of the phase I trial. In the pooled analyses of the two trials, 69 patients were evaluable for response (Phase I, n = 18; Phase II, n = 51). The pooled ORR was 36.2% (40.7% in SyS; 10.0% in MRCLS). Responses occurred across a wide range of MAGE-A4 expression levels, but levels of expression were higher in SyS than in MRCLS. The median duration of response was 52 weeks. The median PFS was 58.3 vs. 11.0 weeks in SyS responders vs. SyS non-responders, respectively [33,45,75,76]. The study is still recruiting SyS patients and further results are awaited.

#### 5.2.3. PRAME

PRAME is a CTA highly expressed at the mRNA and protein level in 70–86% of SyS, frequently showing a strong and diffuse nuclear and cytoplasmatic positivity [65,77]. In addition to looking for PRAME expression in SyS, Luk and coworkers [78] also analyzed the levels of T-cell tumor infiltration and found low T-cell infiltration in 73% of the SyS tumors, intermediate T-cell infiltration in 19%, and high T-cell infiltration in 8% of the tumors. Interestingly, upregulation of HLA-I expression on tumor cells in monophasic and biphasic SyS was associated with infiltration of activated T-cells in specific areas, and such differences in the tumor immune microenvironment can influence responses to immunological therapies [78].

The ACTENGINE IMA203/IMA203CD8 Trial (NCT03686124) is aimed at evaluating TCR-engineered T-cells directed against an HLA-A*02-restricted peptide derived from PRAME after lymphodepletion with fludarabine and cyclophosphamide, with or without the combination of nivolumab in patients with advanced solid tumors. A preliminary report of this study described an ORR of 60% in SyS patients, with three responses out of five treated SyS patients [79]. The results seem of interest also for PRAME, a CTA highly expressed in several tumor types.

The results of the main clinical trials, involving SyS patients, based on the adoptive transfer of engineered immune cells directed against CTA are summarized in Table 1.

**Figure 2:**
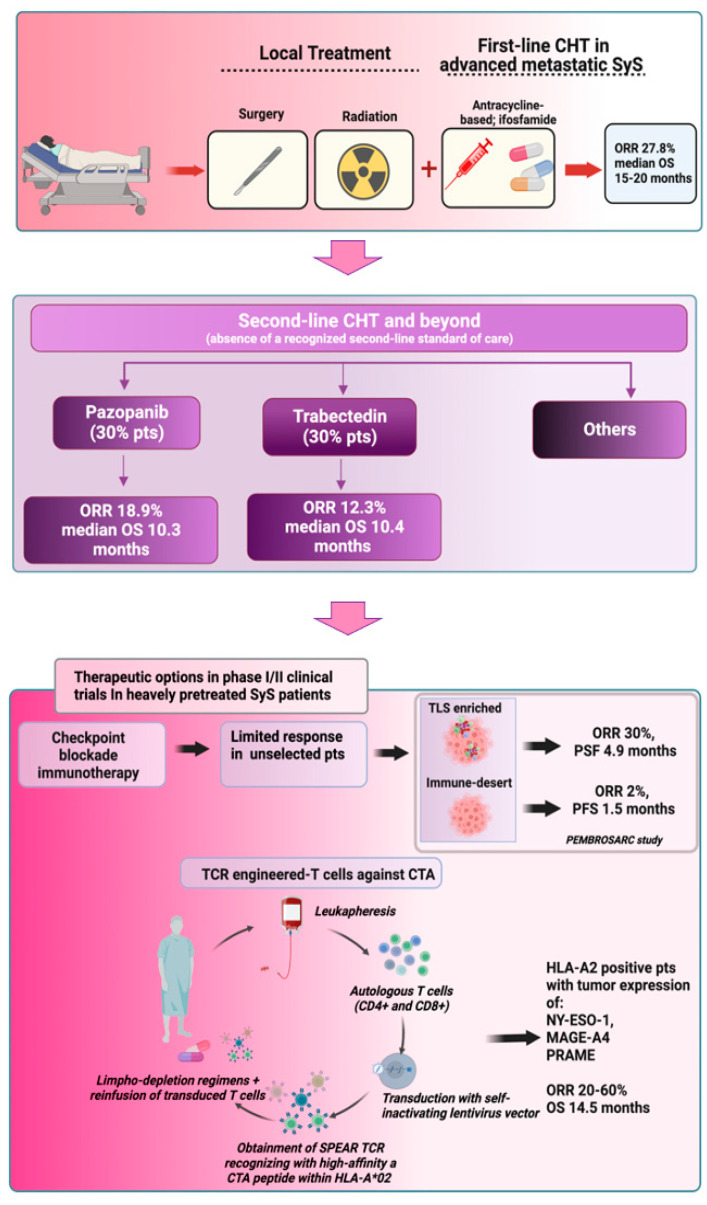
Current therapeutic options for advanced, unresectable, metastatic SyS. After local therapies and front line anthracycline and ifosfamide-based chemotherapy (CHT) [4], patients showing disease relapse undergo second line CHT with decreased ORR and OS [25,30,31]. Immunological therapies under evaluation in phase I/II clinical trials show limited results for ICI in SyS patients, but improved outcomes can derive from the use of tumor immune microenvironment biomarkers for the selection of patients more likely to respond to ICI-based therapies [50,51,62,63]. Higher ORR can be observed in HLA-A2 positive patients receiving TCR-engineered T-cells recognizing cancer/testis antigen (CTA) peptides [66,68,69,70,74,75,76,79] (created with Biorender.com accessed on 25 July 2023).

Outcomes of standard therapeutic options for advanced metastatic SyS and latest immunological therapies under evaluation in clinical trials are illustrated in Figure 2.

Critical issues to the adoptive transfer of TCR engineered T-cells are related to: (i) limitations conferred by HLA-A2 restriction, which restrain the treatment of patients carrying different HLA alleles, (ii) necessity of new technological approaches to enhance TCR specificity and persistence/proliferation of modified TCR-T-cells in the host, (iii) controlled manipulation of the tumor microenvironment to increase clinical responses, and (iv) requirement of qualified expertise for ex-vivo manipulation of T-cells and relatively high costs.

Collectively, data from recent clinical trials based on immunological approaches highlighted new opportunities and challenges for the treatment of advanced SyS. Post-hoc analysis of different clinical trials settled on immunological strategies emphasized that responses were correlated with differences in the tumor immune microenvironment. For treatments with ICIs, a positive correlation was found with the presence of B cells in the tumor infiltrate and for TLS. For therapy involving TCR-engineered T-cell transfer, responses were correlated with a reduced macrophage tumor content. A negative correlation was reported for a low immunogenic tumor microenvironment and the development of resistance due to downregulation of HLA expression.

## 6. Conclusions

Preclinical and clinical investigations on innovative therapeutic options for the treatment of advanced metastatic SyS with a very poor prognosis are a high priority.

In summary, the complex landscape of SyS research and treatment indicates that:

- Current front line treatments, represented by anthracycline and ifosfamide-based chemotherapy, can offer a limited response rate in advanced metastatic SyS, which is below 30%. Response rates further decrease in second line and beyond settings, where in the absence of a recognized standard of care, pazopanib and trabectedin are the most used drugs.
- Epigenetic modifiers, such as HDAC inhibitors and EZH2 inhibitors, have not shown anti-tumor efficacy in SyS in early clinical trials. Other epigenetic drugs, such as BRD9 degraders based on PROTAC technology, are now entering clinical evaluation in SyS.
- Clinical trial results indicate that genetic signatures and biomarkers of the tumor immune microenvironment are highly relevant and predictive of response to both chemotherapy and immunological approaches.
- The use of ICIs in SyS is still challenging. SyS did not emerge as an ICI-sensitive tumor. To improve the response rate, future studies should evaluate ICI-based approaches in selected patients based on tumor immune microenvironment markers.
- Adoptive transfer of TCR-engineered T-cells targeting cancer/testis antigens highly expressed in SyS (NY-ESO-1, MAGE-A4, PRAME) has achieved remarkable ORR in early clinical trials in heavily pretreated advanced metastatic SyS patients with CTA-positive tumors and expressing HLA-A2 aplotype.

Overall, immunological approaches, especially adoptive TCR-engineered T-cell transfer, hold promising results and represent an evolving field with the potential of a high clinical relevance for the management of advanced metastatic SyS. Positive outcomes in early clinical trials highlight the need for further clinical investigations and technological improvements, mainly related to TCR specificity, persistence/proliferation of modified TCR-T-cells in the host, and controlled manipulation of the tumor microenvironment to increase immunogenicity and overcome resistance. Finally, an additional challenge is represented by the extension of these treatments to patients carrying different HLA alleles.

In the field of epigenetic drugs, the PROTAC technology appears to offer a new way to target the epigenetic dysregulation of SyS, and clinical results are awaited.

After further validation of their clinical relevance, entering these technologies into the standard management of advanced SyS patients will represent the next open challenge.

## Figures and Tables

**Table 1:** Main clinical trials, based on adoptive transfer of engineered immune cells, including SyS patients in their cohorts.

Target Antigen	Trial Number	Study Description	Main Results and References
NY-ESO-1	NCT00670748	Pilot phase I study: NY-ESO-1 TCR-T-cells plus IL2 in heavily pretreated metastatic HLA-A*02-positive SyS patients with >50% expression of NY-ESO-1	ORR 61% objective clinical responses in 11/18 SyS patients [66]
	NCT01343043	Pilot phase I study: NY-ESO-1 TCR-T-cells plus IL2 in heavily pretreated metastatic HLA-A*02-positive SyS patients among four cohorts allocated between different levels of NY-ESO-1 expression and lympho-depletion regimens	ORR 20–50% [68,69,70,80]
	NCT03967223	Phase II study: First generation of NY-ESO-1 TCR-T-cells as a first line treatment in advanced metastatic, previously untreated HLA-A*02-positive patients with NY-ESO-1-positive SyS or MRCLS and as a second line treatment after first line anthracycline-based chemotherapy	Active, not recruiting [70]
	NCT04526509	Phase I master protocol of three different next generation NY-ESO-1 TCR-T-cell co-expressing CD8 alpha cell surface receptor, or co-expressing the dominant-negative TGF-beta receptor type II, or engineered using the epigenetically reprogrammed (Epi-R) manufacturing process	Active, not recruiting [70]
	NCT04939701	Phase I/II trial: Human artificial adjuvant vector cells (aAVC) loaded with the CD1d ligand alpha-galactosylceramide and modified to express NY-ESO-1 in combination with pembrolizumab for SyS, MRCLS, ovarian carcinoma, non-small cell lung cancer, and esophageal squamous cell carcinoma	Active, not recruiting [45]
MAGE-A4	NCT03132922	Phase I multi-tumor trial: Afami-cel (carrying TCR specific for a MAGE-A4230−239 peptide, GVYDGREHTV, presented by HLA-A*02) in HLA-A*02+ patients with advanced metastatic MAGE-A4-expressing solid tumors, across nine tumor types including SyS	ORR 44% for SyS and 9% for all other tumors [74]
	NCT04044768	Phase II SPEARHEAD-1 trial: A single arm open-label clinical trial on ADP-A2M4 SPEAR™ T-Cells in HLA-A*02 eligible and MAGE-A4 positive subjects with metastatic or inoperable SyS or MRCLS	ORR 40.7% in SyS [75,76]
PRAME	NCT03686124	The ACTENGINE IMA203/IMA203CD8 trial: TCR-T-cells directed against an HLA-A*02-restricted peptide derived from PRAME after lymphodepletion, with or without nivolumab in patients with advanced solid tumors	ORR 60% in SyS Objective clinical responses in 3/5 SyS patients [79]
